# pTINCR Expression Is Associated with Tumor Aggressiveness but Does Not Independently Predict Prognosis in Head and Neck Cutaneous Squamous Cell Carcinoma

**DOI:** 10.3390/ijms27198443

**Published:** 2026-09-22

**Authors:** Raquel Santos-Juanes, Jorge Santos-Juanes, Noelia Blanco-Agudín, Jesús Merayo-Lloves, Blanca Vivanco, Juana M. García-Pedrero, Juan P. Rodrigo, Iván Fernández-Vega, Cristina Galache

**Affiliations:** 1 Dermatology Service, Hospital Universitario Central de Asturias, 33011 Oviedo, Spain; raquel.santosjuanes@gmail.com (R.S.-J.); galachecristina@uniovi.es (C.G.); 2 Dermatology Area, Department of Medicine, University of Oviedo, 33006 Oviedo, Spain; 3 Grupo de Investigación en Dermatología (GRIDER), Universidad de Oviedo, 33006 Oviedo, Spain; vivancoblanca@gmail.com; 4 Instituto Universitario de Oncología del Principado de Asturias, University of Oviedo, 33011 Oviedo, Spain; juanagp.finba@gmail.com (J.M.G.-P.); jprodrigo@uniovi.es (J.P.R.); 5 Centro de Investigación Biomédica en Red de Cáncer (CIBERONC), Instituto de Salud Carlos III, 28029 Madrid, Spain; 6 Health Research Institute of the Principality of Asturias (ISPA), 33011 Oviedo, Spain; blancoanoelia@uniovi.es (N.B.-A.); merayojesus@uniovi.es (J.M.-L.); 7 Department of Functional Biology, University of Oviedo, 33006 Oviedo, Spain; 8 Instituto Universitario Fernández-Vega (IUFV), Fundación de Investigación Oftalmológica, 33006 Oviedo, Spain; 9 Pathology Service, Hospital Universitario Central de Asturias, 33011 Oviedo, Spain; 10 Pathology Area, Department of Surgery and Medical-Surgical Specialties, University of Oviedo, 33006 Oviedo, Spain; 11 Otolaryngology-Head and Neck Surgery Service, Hospital Universitario Central de Asturias, 33011 Oviedo, Spain; 12 Otolaryngology Area, Department of Surgery and Medical-Surgical Specialties, University of Oviedo, 33006 Oviedo, Spain; 13 Biobank of the Principality of Asturias, 33011 Oviedo, Spain

**Keywords:** cutaneous squamous cell carcinoma, pTINCR, TINCR-encoded microprotein, nodal metastasis, tumor budding, prognosis, immunohistochemistry

## Abstract

Terminal differentiation-induced non-coding RNA (TINCR), initially classified as a long non-coding RNA, encodes a biologically active microprotein (pTINCR) involved in epidermal differentiation and tumor biology. However, the clinicopathological and prognostic relevance of pTINCR expression in cutaneous squamous cell carcinoma (cSCC) remains unclear. We conducted a retrospective case–control study including 116 patients with head and neck cSCC: 58 patients who developed nodal metastasis and 58 non-metastatic controls. pTINCR expression was assessed by immunohistochemistry and analyzed in relation to clinicopathological characteristics and clinical outcomes. Unconditional logistic regression was used to evaluate its association with nodal metastasis. Cox proportional hazards models were additionally used to explore associations with time-to-event outcomes. Negative pTINCR expression was significantly associated with tumor thickness > 6 mm (*p* < 0.001), poorer histological differentiation (*p* = 0.002), and higher tumor stage (*p* = 0.011). In univariate logistic regression, negative pTINCR expression was associated with higher odds of nodal metastasis (OR 2.71, 95% CI 1.27–5.77; *p* = 0.010), but this association was no longer significant after adjustment for established clinicopathological factors (adjusted OR 1.25, 95% CI 0.34–4.59; *p* = 0.736). In Cox analyses, negative pTINCR expression was associated with a shorter time to nodal metastasis (HR 1.88; *p* = 0.017) and higher tumor-specific mortality (HR 2.02; *p* = 0.033) in univariate analyses, but not after multivariable adjustment. Tumor budding remained the strongest independent prognostic factor across the evaluated time-to-event outcomes. Loss of pTINCR expression is associated with adverse histopathological features and nodal metastasis in head and neck cSCC, but it does not provide independent prognostic information beyond established clinicopathological factors. pTINCR may therefore serve as a marker of an aggressive tumor phenotype rather than an independent prognostic biomarker.

## 1. Introduction

Cutaneous squamous cell carcinoma (cSCC) is the second most common skin cancer and arises from epidermal keratinocytes. Although most primary tumors are successfully treated by surgical excision, a subset displays aggressive behavior, including local recurrence, lymph node metastasis, and disease-specific mortality. Prognostic assessment currently relies mainly on established clinical and histopathological characteristics, such as tumor size and thickness, differentiation, perineural invasion, desmoplasia, and tumor budding [1]. Nevertheless, additional biomarkers could improve our understanding of the biological mechanisms underlying cSCC progression and help identify tumors with an aggressive phenotype.

Long non-coding RNAs (lncRNAs), conventionally defined as transcripts longer than 200 nucleotides with limited protein-coding potential, participate in the regulation of tumor initiation, progression, metastasis, and resistance to anticancer therapies [2,3]. Terminal differentiation-induced non-coding RNA (TINCR) was initially classified as an lncRNA and was shown to play an important role in epidermal differentiation and tissue homeostasis [4]. Dysregulation of TINCR has subsequently been described in several malignancies, although its biological function appears to depend on the tissue and molecular context [4].

More recently, Boix et al. demonstrated that TINCR contains a conserved small open reading frame encoding an 87-amino-acid ubiquitin-like microprotein, termed pTINCR [5]. In vitro translation of the full-length transcript generated a protein product of the expected molecular weight, whereas mutation of the translation-initiation codon abolished detectable protein production. Furthermore, pTINCR-specific peptides were identified in human skin by mass spectrometry, and endogenous protein expression was confirmed by Western blotting and immunostaining. Antibody specificity was further supported by loss of the corresponding signal in CRISPR-generated pTINCR-knockout epithelial cells [5,6]. Thus, the protein-coding capacity of TINCR is supported by orthogonal translational, proteomic, and genetic evidence and is not inferred solely from immunohistochemical staining.

Functionally, pTINCR promotes epithelial differentiation, cortical actin organization, and cell–cell adhesion [5]. It interacts with SUMO and CDC42 and enhances CDC42 SUMOylation and activation, thereby promoting an epithelial differentiation program [5]. Experimental evidence also supports a tumor-suppressive role for TINCR in cSCC. Previous studies have implicated the TINCR transcript in the regulation of epigenetic pathways, apoptosis, and autophagy [6,7]. In addition, pTINCR is induced by UV-mediated DNA damage in a TP53-dependent manner, while Tincr-deficient mice develop cutaneous tumors earlier and more frequently following chronic UVB exposure. Restoration of pTINCR expression also suppresses SCC growth in vitro and in vivo [8]. Genomic deletions, protein-disrupting mutations, and reduced pTINCR expression have additionally been identified in human squamous cell carcinomas [8].

Despite this experimental evidence, the clinicopathological and prognostic relevance of pTINCR protein expression in human cSCC remains insufficiently characterized. In particular, it is unclear whether loss of pTINCR is independently associated with lymph node metastasis and adverse clinical outcomes or whether it predominantly reflects established histopathological characteristics of tumor aggressiveness.

Therefore, the primary aim of this study was to evaluate the association between pTINCR protein expression and lymph node metastasis in patients with head and neck cSCC. We also examined its relationship with established clinicopathological characteristics, including tumor thickness, differentiation, pathological stage, perineural invasion, desmoplastic growth, and tumor budding. As secondary exploratory objectives, we assessed the association of pTINCR expression with time to nodal metastasis, tumor-specific mortality, and all-cause mortality.

## 2. Results

### 2.1. Patient Characteristics and pTINCR Expression

A total of 116 White patients were included in the study (Table 1), of whom 89 (76.7%) were male. The mean age of the overall study population was 78.7 ± 8.3 years. Patients with pTINCR-negative tumors had a mean age of 79.11 ± 7.73 years, compared with 78.41 ± 8.87 years among those with pTINCR-positive tumors.

Regarding pTINCR expression, 52 patients (44.8%) had pTINCR-negative tumors, whereas 64 (55.2%) had pTINCR-positive tumors (Figure 1). No significant differences were observed between the pTINCR-negative and pTINCR-positive groups in sex distribution (76.9% vs. 76.6%; *p* = 1.000) or mean age (79.11 ± 7.73 vs. 78.41 ± 8.87 years; *p* = 0.713).

With respect to tumor burden, pTINCR-negative tumors tended to be thicker than pTINCR-positive tumors (10.02 ± 6.50 vs. 8.02 ± 6.49 mm), although the difference was not statistically significant (*p* = 0.101). However, when tumor thickness was analyzed categorically, tumors > 6 mm were significantly more frequent in the pTINCR-negative group (69.2% vs. 37.5%; *p* < 0.001).

Horizontal tumor diameter did not differ significantly between the groups, either as a continuous variable (*p* = 0.275) or after dichotomization at 20 mm (*p* = 0.700).

A significant association was observed between pTINCR expression and histological differentiation (*p* = 0.002). Moderately and poorly differentiated tumors were more frequent in the pTINCR-negative group, whereas well-differentiated tumors predominated in the pTINCR-positive group. Similarly, pTINCR expression was significantly associated with pathological tumor stage (*p* = 0.011), with pTINCR loss being associated with more advanced disease.

No significant differences were observed in desmoplastic growth, ear location, inflammation, synchronous cSCCs, immunosuppression, perineural invasion, or lymphovascular invasion.

Tumor budding showed a non-significant trend toward a higher prevalence in pTINCR-negative tumors (55.8% vs. 39.1%; *p* = 0.093). A similar trend was observed for tumors with ≥5 buds (28.8% vs. 14.1%; *p* = 0.066).

### 2.2. Logistic Regression and Sensitivity Analyses

In the primary univariable logistic regression analysis, negative pTINCR expression was associated with higher odds of nodal metastasis (OR 2.71; 95% CI, 1.27–5.77; *p* = 0.010). However, this association was no longer statistically significant after adjustment for tumor thickness, horizontal tumor size, desmoplastic growth, perineural invasion, tumor bud-ding, and histological differentiation (adjusted OR 1.25; 95% CI, 0.34–4.59; *p* = 0.736).

To evaluate the robustness of the binary cutoff, a sensitivity analysis was performed using the original three-category pTINCR score. Of the 116 tumors, 52 had a score of 0, 53 had a score of 1, and 11 had a score of 2. The three-category analysis confirmed significant asso-ciations between pTINCR score and nodal metastasis (*p* = 0.029), tumor thickness (*p* = 0.003), histological differentiation (*p* = 0.004), and pathological tumor stage (*p* = 0.022).

When pTINCR score was treated as an ordinal variable, increasing pTINCR expression was associated with lower odds of nodal metastasis in the univariable analysis (OR per one-category increase 0.53; 95% CI, 0.29–0.95; *p* = 0.034). This association was attenuated and was no longer statistically significant after adjustment for the same clinicopathological factors (adjusted OR 0.72; 95% CI, 0.25–2.07; *p* = 0.540). Thus, the sensitivity analyses were consistent with the primary analysis: lower pTINCR expression was associated with nodal metastasis in the unadjusted analysis but did not provide independent information after clinicopathological adjustment.

pTINCR-negative tumors showed a higher prevalence of tumor budding than pTINCR-positive tumors (55.8% vs. 39.1%; OR 1.97; 95% CI, 0.94–4.13; *p* = 0.074). High-grade budding, defined as ≥5 tumor buds, was also more frequent in pTINCR-negative tumors (28.8% vs. 14.1%; OR 2.48; 95% CI, 0.98–6.25; *p* = 0.055). Alt-hough these associations did not reach conventional statistical significance, both showed a consistent trend toward increased tumor budding in tumors lacking pTINCR expression.

### 2.3. Time-to-Event Analyses

In the secondary univariable Cox regression analyses, negative pTINCR expression was associated with a shorter time to nodal metastasis (HR 1.88; 95% CI, 1.18–3.17; *p* = 0.017) and higher tumor-specific mortality (HR 2.02; 95% CI, 1.06–3.86; *p* = 0.033). The association with all-cause mortality did not reach statistical significance (HR 1.38; 95% CI, 0.91–2.09; *p* = 0.125).

In exploratory Cox models adjusted specifically for the presence of tumor budding, the association between negative pTINCR expression and time to nodal metastasis was attenuated and was no longer statistically significant (HR 1.53; 95% CI, 0.90–2.60; *p* = 0.112). A similar attenuation was observed for tumor-specific mortality (HR 1.58; 95% CI, 0.82–3.03; *p* = 0.173). For all-cause mortality, negative pTINCR expression remained nonsignificant after adjustment for tumor budding (HR 1.24; 95% CI, 0.81–1.90; *p* = 0.316). In contrast, tumor budding remained strongly associated with time to nodal metastasis (HR 7.59; 95% CI, 3.96–14.55; *p* < 0.001) and tumor-specific mortality (HR 4.99; 95% CI, 2.34–10.66; *p* < 0.001).

Several clinicopathological variables were significantly associated with the evaluated outcomes in the univariable Cox analyses. Tumor thickness, horizontal tumor size, desmoplastic growth, perineural invasion, tumor budding, and histological differentiation were associated with time to nodal metastasis. Tumor thickness, horizontal tumor size, perineural invasion, and tumor budding were associated with tumor-specific mortality. Age, tumor thickness, horizontal tumor size, perineural invasion, and tumor budding were associated with all-cause mortality.

After multivariable adjustment, pTINCR expression did not retain independent prognostic significance for time to nodal metastasis (HR 1.02; 95% CI, 0.57–1.82; *p* = 0.692), tumor-specific mortality (HR 0.96; 95% CI, 0.56–1.63; *p* = 0.876), or all-cause mortality (HR 0.76; 95% CI, 0.47–1.22; *p* = 0.258).

Tumor thickness remained independently associated with time to nodal metastasis (HR 2.88; 95% CI, 1.34–6.17; *p* = 0.007) and tumor-specific mortality (HR 4.13; 95% CI, 1.60–10.65; *p* = 0.003). Horizontal tumor size was independently associated with tumor-specific mortality (HR 2.33; 95% CI, 1.07–5.35; *p* = 0.046) and all-cause mortality (HR 2.34; 95% CI, 1.27–4.33; *p* = 0.007).

Tumor budding was the strongest independent prognostic factor and remained significantly associated with time to nodal metastasis (HR 5.97; 95% CI, 2.99–11.86; *p* < 0.001), tumor-specific mortality (HR 4.72; 95% CI, 2.08–10.72; *p* < 0.001), and all-cause mortality (HR 1.99; 95% CI, 1.23–3.20; *p* = 0.005). Age was independently associated with tumor-specific mortality (HR 1.07; 95% CI, 1.02–1.12; *p* = 0.008) and all-cause mortality (HR 1.08; 95% CI, 1.04–1.12; *p* < 0.001) (Table 2).

### 2.4. Kaplan–Meier Analyses

The median follow-up duration, estimated using the reverse Kaplan–Meier method, was 108 months for the overall cohort, 107 months for patients with pTINCR-negative tumors, and 108 months for those with pTINCR-positive tumors. Overall, 58 patients developed nodal metastasis, including 33 in the pTINCR-negative group and 25 in the pTINCR-positive group. A total of 38 patients died from cSCC, including 22 with pTINCR-negative tumors and 16 with pTINCR-positive tumors. Ninety patients died from any cause: 42 in the pTINCR-negative group and 48 in the pTINCR-positive group.

Kaplan–Meier analysis showed significant differences according to pTINCR expression in metastasis-free survival (*p* = 0.015) and tumor-specific survival (*p* = 0.029), with pTINCR-negative tumors showing poorer outcomes. No significant difference in overall survival was observed between the pTINCR-positive and pTINCR-negative groups (Figure 2).

## 3. Discussion

The present study provides clinical evidence complementing previous experimental findings on the role of pTINCR in cutaneous squamous cell carcinoma (cSCC) [6,7]. Loss of pTINCR expression was significantly associated with several histopathological features of tumor aggressiveness, including tumor thickness > 6 mm, poorer histological differentiation, and more advanced pT stage. Although pTINCR negativity was associated with an increased risk of nodal metastasis and tumor-specific mortality in univariate time-to-event analyses, it did not retain independent prognostic significance after multivariable adjustment. This suggests that the observed prognostic association may largely reflect the relationship between pTINCR loss and established pathological risk factors rather than an independent effect on clinical outcomes.

Among the variables analyzed, tumor budding emerged as one of the strongest independent predictors of nodal metastasis and tumor-specific mortality. This finding is consistent with growing evidence supporting tumor budding as a robust histopathological prognostic marker in cSCC [1]. Notably, pTINCR-negative tumors showed a consistently higher prevalence of both any tumor budding and high-grade budding, although these associations narrowly failed to reach conventional statistical significance. In exploratory models adjusted specifically for tumor budding, the associations between pTINCR loss and both nodal metastasis and tumor-specific mortality were attenuated and no longer statistically significant. These findings suggest that the relationship between pTINCR loss and adverse outcomes may be partly explained by the greater prevalence of an invasive budding phenotype among pTINCR-negative tumors. Biologically, loss of pTINCR-associated epithelial differentiation could facilitate the acquisition of this phenotype, thereby linking reduced pTINCR expression to tumor progression. However, because no formal mediation analysis was performed and the study was retrospective, tumor budding cannot be considered a demonstrated mediator of this association.

These clinical observations are consistent with experimental findings previously reported by our group showing that pTINCR functions as a tumor-suppressive microprotein in SCC [8]. pTINCR is a highly conserved ubiquitin-like microprotein encoded by the TINCR transcript and is involved in epithelial differentiation and the TP53-dependent response to UV-induced DNA damage. TINCR-deficient mice developed cSCC earlier and more frequently following chronic UVB exposure, whereas restoration of pTINCR expression suppressed tumor growth both in vitro and in vivo. Recurrent genomic deletions, loss-of-function mutations, and reduced pTINCR expression in human SCC provide further support for its tumor-suppressive role [8].

Additional studies have identified potentially relevant functions of the TINCR transcript. At the RNA level, TINCR has been implicated in epigenetic regulation through the recruitment of DNMT1 [6]. In cSCC cells, TINCR also participates in ALA-PDT-induced apoptosis and autophagy through the ERK1/2–SP3 pathway [6]. In oral squamous cell carcinoma, reduced TINCR expression has been associated with poor differentiation, lymph node metastasis, and unfavorable survival, while experimental findings suggest that TINCR suppresses epithelial-to-mesenchymal transition and invasion through inhibition of the JAK2/STAT3 pathway [9]. Collectively, these mechanisms provide a plausible biological explanation for the association between reduced pTINCR expression and aggressive clinicopathological features in cSCC. Nevertheless, the present observational study did not directly investigate these mechanisms.

The biological relevance of TINCR may also be considered within the broader context of the cutaneous response to ultraviolet radiation. UV-responsive lncRNAs participate in regulatory networks involving keratinocyte apoptosis, oxidative stress, inflammation, and epidermal differentiation, thereby contributing to epidermal homeostasis following environmental injury [10]. Given that chronic ultraviolet exposure is the principal etiological factor for cSCC, these observations provide a broader biological context for the relationship between dysregulation of differentiation-associated pathways and aggressive tumor behavior.

Although accumulating evidence supports a tumor-suppressive function of TINCR in squamous epithelial malignancies [2,11], its biological role appears to be highly tissue- and context-dependent. Increased TINCR expression has been reported in gastric, esophageal, bladder, and breast carcinomas, whereas reduced expression has been described in oral squamous cell carcinoma, colorectal cancer, prostate cancer, glioma, retinoblastoma, and metastatic melanoma [12,13,14,15,16,17]. These apparently contradictory findings suggest that TINCR may exert oncogenic or tumor-suppressive effects depending on tissue-specific transcriptional programs, molecular interactions, and cellular context. Moreover, some of these effects may be mediated by the TINCR transcript, whereas others may depend on its encoded microprotein.

Our findings should therefore be interpreted within the emerging paradigm that some transcripts previously classified as long non-coding RNAs encode biologically active microproteins. More than 80 lncRNA-derived microproteins have now been experimentally validated and implicated in processes including proliferation, DNA damage repair, metabolism, cell-cycle regulation, and differentiation. pTINCR is among the best-characterized examples and illustrates how a single transcript may exert distinct RNA-dependent and protein-dependent functions. Mechanistically, pTINCR promotes cortical actin organization, enhances CDC42 SUMOylation, strengthens epithelial cell–cell adhesion, and is inversely associated with cell-cycle-related gene expression [5,8,18]. These functions provide a plausible mechanistic basis for the association observed in our cohort between loss of pTINCR expression and a poorly differentiated, invasive, and clinically aggressive cSCC phenotype.

Several limitations should be acknowledged. First, this was a retrospective, single-center case–control study with a relatively limited sample size, which may have reduced the precision and statistical power of the multivariable analyses. Moreover, no independent external validation cohort was available. This is particularly relevant when evaluating the potential value of pTINCR as a biomarker, as its reproducibility, generalizability, and clinical utility cannot be established from a single cohort. Therefore, the present findings should be regarded as exploratory and hypothesis-generating and require validation in larger, independent, multicenter cohorts. The selection of equal numbers of metastatic cases and non-metastatic controls was appropriate for evaluating associations with metastasis but limits estimation of absolute risks and requires cautious interpretation of the secondary time-to-event analyses. Second, only 11 tumors showed strong, homogeneous pTINCR expression, limiting the precision of analyses based on the original three-category score. Nevertheless, the ordinal sensitivity analysis produced findings consistent with the primary binary analysis. Third, immunohistochemical assessment using tissue microarrays may not fully represent intratumoral heterogeneity, despite the evaluation of multiple tumor cores. Finally, observational design does not permit causal or mechanistic conclusions regarding the role of pTINCR in tumor progression. The study did not include functional intervention experiments, direct evaluation of molecular binding partners, or therapeutic validation. Independent clinical cohorts and studies integrating transcriptomic, proteomic, and functional approaches will be necessary to confirm these findings and clarify the respective contributions of the TINCR transcript and the encoded microprotein.

Taken together, our findings indicate that loss of pTINCR expression identifies a more aggressive clinicopathological phenotype in head and neck cSCC but does not provide independent prognostic information beyond established histopathological risk factors. Tumor budding remained the strongest independent predictor of the evaluated outcomes and may partly account for the univariable associations between pTINCR loss and adverse clinical outcomes. These results are consistent with the biological relevance of pTINCR in epithelial differentiation and cSCC progression and provide a clinical basis for further mechanistic investigation. However, they do not demonstrate therapeutic efficacy or direct translational applicability, which will require validation in dedicated functional and preclinical studies [11,19].

## 4. Materials and Methods

### 4.1. Patients and Procedures

We searched the electronic database of the Department of Pathology at Hospital Universitario Central de Asturias to identify all patients who developed histologically confirmed nodal metastases from head and neck cutaneous squamous cell carcinoma (cSCCHN) between 1998 and 2017. Electronic medical records were reviewed to ascertain the outcomes of interest. The study included 58 patients with primary cSCCHN who developed nodal metastases and 58 randomly selected controls with cSCCHN who remained metastasis-free during at least 5 years of follow-up. All tumors were treated by conventional surgical excision; patients with positive surgical margins were excluded, and none received postoperative adjuvant therapy. The study was conducted in accordance with the Declaration of Helsinki and approved by the Drug Research Ethics Committee of the Principality of Asturias (CEImPA; approval code 2022-439; approval date: 24 October 2022). The study was conducted and reported in accordance with the Strengthening the Reporting of Observational Studies in Epidemiology guidelines for case–control studies. Clinical data were collected retrospectively.

### 4.2. Histopathological Evaluation

Hematoxylin and eosin-stained slides were reviewed for each sample. The following histopathological features were recorded:

- Maximum tumor diameter;
- Tumor thickness, categorized as ≤6 mm or >6 mm (no tumor measured <2 mm);
- Anatomical level according to Clark’s classification;
- Histopathological differentiation, classified as well (grade 1), moderately (grade 2), or poorly differentiated (grade 3);
- Presence of desmoplasia;
- Perineural or lymphovascular invasion; and
- Presence and number of tumor buds.

Tumor budding. Tumor budding was defined as isolated single cells or small clusters of up to four cells scattered within the stroma ahead of the invasive tumor front. The budding index was classified as low (<5 buds) or high (≥5 buds) [7].

### 4.3. Tissue Microarray Construction

Tissue selection and core extraction. Morphologically representative areas were selected from each paraffin-embedded tumor block. Three 1 mm tissue cores were obtained from each tumor using a previously described method [20].

TMA composition. Five tissue microarrays (TMAs) were constructed, each incorporating three cores from the relevant specimens across the cohort of 116 cSCCHNs. Each TMA also included three normal skin cores as internal controls.

### 4.4. Antibody Validation and Immunohistochemistry

A previously characterized custom rabbit polyclonal antibody directed against the pTINCR microprotein was used [5,8]. The antibody was generated against immunogenic pTINCR peptide sequences, and its specificity was previously validated using CRISPR-generated pTINCR-knockout HaCaT and MCF7 cells. The endogenous pTINCR signal detected at the expected molecular weight in wild-type cells was lost in pTINCR-knockout cells, and loss of staining was independently confirmed by immunostaining. Previous tissue analyses also included non-expressing tissues as negative controls [5]. Negative immunohistochemical controls performed by omission of the primary antiserum showed no detectable staining [8].

TMA sections were cut at 3 µm and mounted on Flex IHC microscope slides (Dako, Glostrup, Denmark). Sections were deparaffinized in xylene, rehydrated through graded alcohols, and subjected to heat-induced antigen retrieval using EnVision FLEX Target Retrieval Solution, High pH (Dako). Staining was performed at room temperature on a Dako Autostainer Plus using the custom rabbit polyclonal anti-pTINCR antibody at a 1:1000 dilution. Immunoreactivity was visualized using the Dako EnVision FLEX+ Visualization System with diaminobenzidine as the chromogen. Sections were counterstained with hematoxylin. Normal skin was included as a positive tissue control, whereas sections processed without the primary antibody served as negative controls.

TINCR expression scoring. pTINCR expression was assessed using a semiquantitative system based on cytoplasmic staining intensity:

- Score 0 (negative): no staining.
- Score 1 (weak to moderate): focal cytoplasmic staining in tumor areas.
- Score 2 (strong): intense, homogeneous cytoplasmic staining in tumor areas.

Interobserver concordance exceeded 95%. For statistical analyses, scores of 1–2 were classified as positive TINCR expression, whereas a score of 0 was classified as negative expression or loss of TINCR expression. This cutoff was selected because the biological hypothesis focused on complete loss of pTINCR expression, in accordance with its proposed tumor-suppressive function. A score of 0 therefore represented complete absence of detectable pTINCR, whereas scores of 1 and 2 indicated preserved protein expression. Scores 1 and 2 were combined because only 11 tumors showed strong, homogeneous expression, which limited the precision of separate estimates for this category.

### 4.5. Statistical Analysis

#### Descriptive Statistics and Outcomes

Baseline demographic, clinical, and histopathological characteristics were summarized using standard descriptive statistics. Continuous variables are presented as mean ± standard deviation (SD) or as median and interquartile range, as appropriate. Categorical variables are reported as absolute frequencies and percentages. Comparisons according to pTINCR expression were performed using Student’s *t*-test or the Mann–Whitney U test for continuous variables, depending on their distribution, and Pearson’s chi-square test or Fisher’s exact test for categorical variables, as appropriate.

Given the retrospective case–control design, unconditional binary logistic regression was used as the primary analytical approach to evaluate the association between pTINCR expression and lymph node metastasis. Nodal metastasis status was the dependent variable and was coded as absent (0) or present (1). pTINCR expression was entered as a binary variable, with positive expression as the reference category. Crude and adjusted odds ratios (ORs) with 95% confidence intervals (CIs) were calculated.

To reduce the risk of overfitting, the multivariable logistic regression model was restricted to pTINCR expression and established histopathological factors showing prognostic relevance in the univariable analyses: tumor thickness, horizontal tumor size, desmoplastic growth, perineural invasion, tumor budding, and histological differentiation. Age and sex were not retained because they were not associated with nodal metastasis in the univariable analyses. The final model therefore included seven parameters for 58 metastatic events. Tumor thickness and horizontal tumor size were entered as categorical variables using the prespecified cutoffs of >6 mm and >20 mm, respectively. pTINCR expression, desmoplastic growth, perineural invasion, and tumor budding were treated as binary variables, whereas histological differentiation was entered as an ordinal variable.

Multicollinearity was assessed using variance inflation factors (VIFs). All VIF values were below 2 (maximum VIF, 1.72), indicating no relevant multicollinearity among the included covariates. Model calibration was evaluated using the Hosmer–Lemeshow goodness-of-fit test and was considered adequate (χ^2^ = 3.11; *p* = 0.875).

The primary immunohistochemical analysis classified score 0 as negative pTINCR expression and scores 1–2 as positive expression. To assess the robustness of this cutoff, sensitivity analyses were performed using the original three-category score (0, 1, and 2) and by entering the score as an ordinal variable in logistic regression. In the ordinal analysis, the OR represents the change in the odds of nodal metastasis associated with each one-category increase in pTINCR expression.

Time-to-event analyses were performed as secondary exploratory analyses. Time to nodal metastasis was calculated from the date of diagnosis of the primary tumor to the date of histologically confirmed nodal metastasis; patients who did not develop metastasis were censored at their last follow-up. Tumor-specific survival was calculated from the date of primary tumor diagnosis to death attributable to cSCC. Patients who were alive or died from another cause were censored at their last follow-up or date of death, respectively. Overall survival was calculated from the date of primary tumor diagnosis to death from any cause, with surviving patients censored at their last follow-up. Median follow-up duration was estimated using the reverse Kaplan–Meier method.

Kaplan–Meier curves were used to estimate nodal metastasis-free survival, tumor-specific survival, and overall survival according to pTINCR expression. Differences between the pTINCR-positive and pTINCR-negative groups were assessed using the log-rank test. Numbers at risk were reported below the Kaplan–Meier curves.

Cox proportional hazards regression was used to evaluate associations between pTINCR expression, clinicopathological variables, and each time-to-event outcome. Crude and adjusted hazard ratios (HRs) with 95% CIs were calculated. pTINCR expression was included in all multivariable Cox models. Additional covariates were selected according to their established clinicopathological relevance, their association with the corresponding outcome in the univariable analysis, and the number of observed events, to limit model overfitting. Age was analyzed as a continuous variable; tumor thickness and horizontal tumor size were entered using the categories described above; pTINCR expression, sex, desmoplastic growth, perineural invasion, and tumor budding were treated as binary variables; and histological differentiation was treated as an ordinal variable. Exploratory Cox models adjusted specifically for tumor budding were also fitted to assess whether tumor budding could partly account for the univariable associations between pTINCR loss and adverse time-to-event outcomes.

All statistical tests were two-sided, and *p* values < 0.05 were considered statistically significant. Given the exploratory nature of the study, no adjustment for multiple comparisons was performed. Statistical analyses were conducted using IBM SPSS Statistics for Windows, version 27.0 (IBM Corp., Armonk, NY, USA).

## 5. Conclusions

Loss of pTINCR expression identifies a more aggressive clinicopathological phenotype in head and neck cSCC but does not independently predict nodal metastasis after adjustment for established risk factors. These findings support the potential biological relevance of pTINCR; however, any diagnostic, prognostic, or therapeutic application remains hypothetical. Additional functional and preclinical studies are required to determine whether pTINCR-related pathways could represent future therapeutic targets.

## Figures and Tables

**Figure 1:**
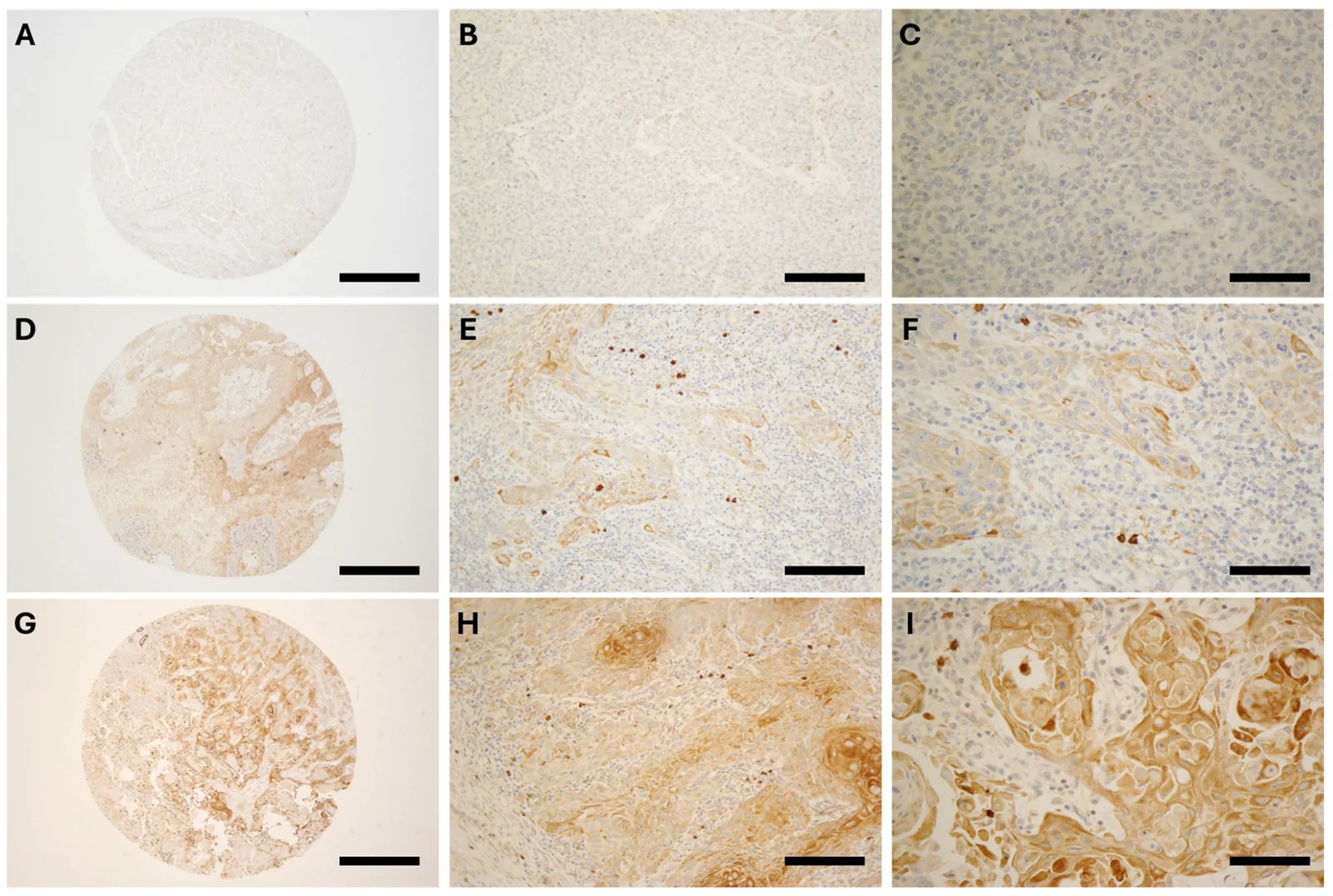
Representative immunohistochemical patterns of pTINCR expression in cutaneous squamous cell carcinoma (cSCC). (**A**–**C**) Negative pTINCR expression, showing absent or minimal immunoreactivity. (**D**–**F**) Partially positive pTINCR expression, characterized by focal and heterogeneous immunoreactivity. (**G**–**I**) Positive pTINCR expression, showing diffuse immunoreactivity in tumor cells. Low-power images of 1-mm tissue microarray (TMA) cores are shown in (**A**,**D**,**G**) (2× objective); higher-magnification images are shown in (**B**,**E**,**H**) (20× objective) and in (**C**,**F**,**I**) (40× objective). Scale bars: 300 μm (**A**,**D**,**G**), 100 μm (**B**,**E**,**H**), and 50 μm (**C**,**F**,**I**).

**Figure 2:**
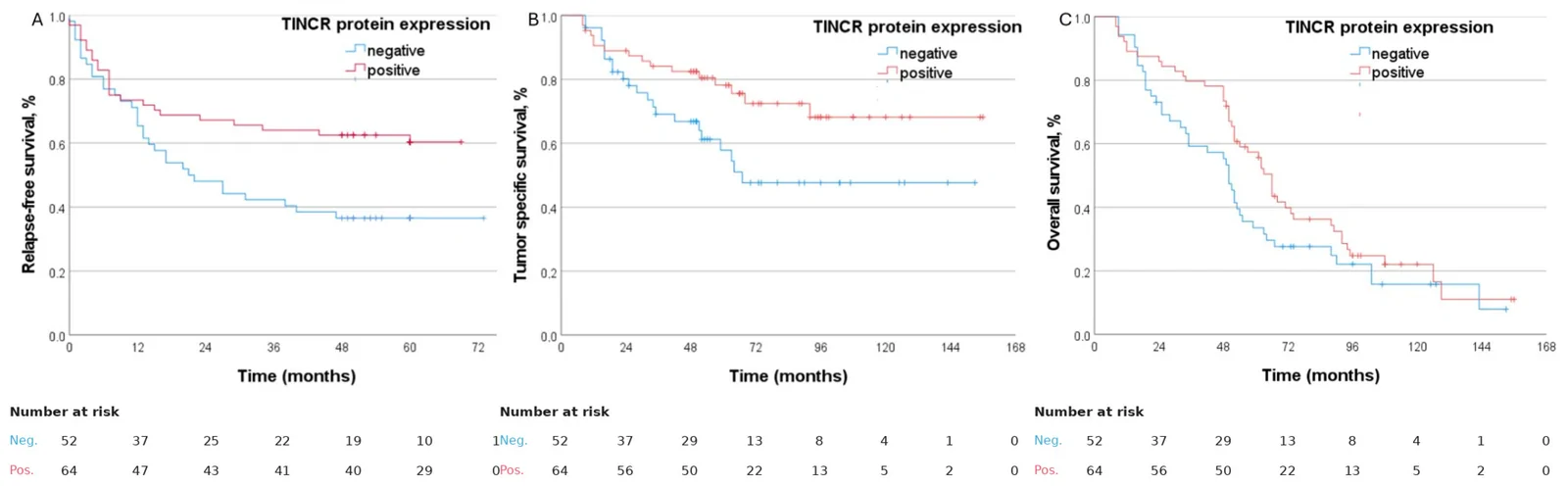
Kaplan–Meier analyses according to pTINCR expression in head and neck cutaneous squamous cell carcinoma. Numbers at risk are shown below each panel. (**A**) Nodal metastasis-free survival was significantly lower in patients with pTINCR-negative tumors than in those with pTINCR-positive tumors (*p* = 0.015; 33 vs. 25 metastatic events, respectively). (**B**) Tumor-specific survival was significantly lower in the pTINCR-negative group (*p* = 0.029; 22 vs. 16 cSCC-specific deaths). (**C**) No statistically significant difference in overall survival was observed between the groups (42 vs. 48 deaths from any cause). Differences were assessed using the log-rank test.

**Table 1:** Association between pTINCR expression and clinicopathological features of patients with primary cutaneous squamous cell carcinoma.

		pTINCR Expression		
Clinicopathological Characteristics	N = 116	Negative N = 52; N (44.8%)	Positive N = 64; N (55.2%)	*p*
Sex, male	89	40 (76.9)	49 (76.6)	1.000
Age, mean ± SD, (min–max)	78.74 ± 8.32 (50–97)	79.11 ± 7.73 (51–93)	78.41 ± 8.87 (50–97)	0.713
Tumor thickness, mm, mean ± SD	8.91 ± 6.54	10.02 ± 6.50	8.02 ± 6.49	0.101
>6 mm	60	36 (69.2)	24 (37.5)	<0.001
Tumor horizontal size, mm, mean ± SD	21.56 ± 13.89	23.17 ± 15.77	20.25 ± 12.14	0.275
>20 mm	42	20 (38.5)	22 (34.4)	0.700
Tumor differentiation				0.002
Well differentiated	61 (52.6%)	18 (34.6)	43 (67.2)	
Moderately differentiated	48 (41.4%)	29 (55.8)	19 (29.7)	
Poorly differentiated	7 (6.0%)	5 (9.6)	2 (3.1)	
Desmoplastic growth	21	12 (23.1)	9 (14.1)	0.233
Tumor site, ear	26	12 (23.1)	14 (21.9)	1.000
pTNM, AJCC 8th edition				0.011
pT1	64 (55.2%)	22 (42.3%)	42 (65.6%)	
pT2	36 (31.0%)	18 (34.6%)	18 (28.1%)	
pT3	16 (13.8%)	12 (23.1%)	4 (6.3%)	
Inflammation				0.494
None	29	14 (26.9)	15 (23.4)	
Mild	73	30 (57.7)	43 (67.2)	
Strong	14	8 (15.4)	6 (9.4)	
Other SCC	38	15 (28.8)	23 (35.9)	0.435
Immunosuppression				0.287
Yes	16 (13.8%)	5 (9.6)	11 (17.2)	
No	100 (86.2%)	47 (90.4)	53 (82.8)	
Perineural invasion				0.176
Yes	16 (13.8%)	10 (19.2)	6 (9.4)	
No	100 (86.2%)	42 (80.8)	58 (90.6)	
Lymphovascular invasion				1.000
Yes	3 (2.6%)	1 (1.9)	2 (3.1)	
No	113 (97.4%)	51 (98.1)	62 (96.9)	
Tumor buds present				0.093
Yes	54	29 (55.8)	25 (39.1)	
No	62	23 (44.2)	39 (60.9)	
≥5 tumor buds				0.066
Yes	24	15 (28.8)	9 (14.1)	
No	92	37 (71.2)	55 (85.9)	

**Table 2:** Univariate and multivariate models for the effect of pTINCR expression on nodal metastasis, tumor mortality and all-cause mortality.

	Metastasis		Tumor Mortality		All-Cause Mortality	
pTINCR Negative	HR (95% CI)	*p*	HR (95% CI)	*p*	HR (95% CI)	*p*
			**Univariate**			
pTINCR negative	1.88 (1.18–3.17)	0.017	2.02 (1.06–3.86)	0.033	1.38 (0.91–2.09)	0.125
Age per year	1.00 (0.972–1.03)	0.836	1.05 (1.00–1.09)	0.049	1.06 (1.03–1.09)	<0.001
Sex male	1.18 (0.63–2.24)	0.597	1.07 (0.49–2.34)	0.858	0.80 (0.50–1.29)	0.367
Tumor thickness > 6	5.26 (2.82–9.81)	<0.001	6.09 (2.67–13.88)	<0.001	1.63 (1.07–2.48)	0.021
Tumor horizontal > 20	3.99 (2.35–6.76)	<0.001	3.96 (2.07–7.59)	<0.001	2.21 (1.44–3.40)	<0.001
Desmoplastic growth	2.47 (1.40–4.37)	0.002	1.93 (0.94–3.98)	0.075	1.25 (0.72–2.15)	0.423
Perineural invasion	3.60 (1.97–6.61)	<0.001	2.30 (1.5–5.049	0.038	1.84 (1.01–3.34)	0.045
Tumor budding	7.93 (4.15–15.14)	<0.001	5.38 (2.54–11.40)	<0.001	1.94 (1.28–2.94)	0.002
Tumor differentiation	2.34 (1.56–3.50)	<0.001	1.53 (0.95–2.46)	0.082	1.21 (0.87–1.70)	0.250
			**Multivariate**			
pTINCR negative	1.12 (0.86–1.96)	0.692	0.96 (0.56–1.63)	0.876	0.76 (0.47–1.22)	0.258
Age per year	0.99 (0.96–1.03)	0.882	1.07 (1.02–1.12)	0.008	1.08 (1.04–1.12)	<0.001
Tumor thickness > 6	2.88 (1.34–6.17)	0.007	4.13 (1.60–10.65)	0.003	1.03 (0.99–1.07)	0.131
Tumor horizontal > 20	1.60 (0.83–3.11)	0.160	2.33 (1.07–5.35)	0.046	2.34 (1.27–4.33)	0.007
Desmoplastic growth	0.82 (0.40–1.69)	0.586	0.83 (0.35–1.95)	0.670	0.77 (0.41–1.44)	0.419
Perineural invasion	1.33 (0.64–2.80)	0.445	0.53 (0.22–1.29)	0.163	0.64 (0.32–1.31)	0.228
Tumor buds	5.97 (2.99–11.86)	<0.001	4.72 (2.08–10.72)	<0.001	1.99 (1.23–3.2)	0.005
Tumor differentiation	1.39 (0.91–2.12)	0.123	0.90 (0.52–1.53)	0.697	0.92 (0.61–1.41)	0.706

## Data Availability

The raw data supporting the conclusions of this article will be made available by the authors on request.
